# Spontaneous electrical activity recorded from the aphid central nervous system

**DOI:** 10.1007/s10158-012-0141-x

**Published:** 2012-09-21

**Authors:** Dan-Thanh T. Nguyen, Melissa J. Blacker, James A. Goodchild

**Affiliations:** Department of Biological Sciences, Syngenta Crop Protection, Jealott’s Hill Research Station, Bracknell, Berkshire, RG42 6EY UK

**Keywords:** Aphids, Electrophysiology, Extracellular recording, Imidacloprid, *Tuberolachnus salignus*

## Abstract

Whilst many classes of insecticides target the insect central nervous system (CNS), their effects in the CNS of pest aphids have not been demonstrated. In this report, we describe an electrophysiological method for recording spontaneous neuronal activity from the giant willow aphid (*Tuberolachnus salignus*). Using extracellular recording electrodes and two analysis methods (threshold and template search), spontaneous spike activity was shown to exhibit sensitivity to the neuroexcitatory insecticide imidacloprid. This method allows changes in the frequency of action-potentials to be monitored during direct bath exposure to chemical agents, enabling a means of assessing and comparing neurotoxic effects of insecticides in a previously inaccessible superfamily of pest insects.

## Introduction

Aphids and other sap-sucking insects are important pests in agriculture and horticulture causing widespread damage to crops and ornamentals through feeding and transmission of plant viruses (van Emden and Harrington 2007). As such, they are important commercial targets in the crop-protection industry (Dedryver et al. 2010). The development of new insecticides begins with the identification of new lead compounds, which can be identified by screening directly against target pest species. Unfortunately, the performance of a chemical in a whole organism is far from simple, arising from a complex myriad of factors including uptake, translocation, metabolic stability—both in the target organism and its host plant—and the physiological role of the target protein. In order to determine the contribution of these individual factors to the overall activity, they often need to be experimentally assessed separately. Electrophysiology is commonly used in this context because it removes some of the biomobility factors, allowing a more direct visualisation of the effects of exploratory compounds at their target organs or receptors. Recordings of spontaneous activity in complex neuronal circuits can provide a useful measure of neurotoxicity where obtaining voltage-clamped patch recordings from isolated or identified neurones have proven particularly difficult.

Electrophysiological techniques in aphids have been in common use over the last 30 years although the number of applications have been somewhat limited: Electrical penetration graphs were developed as a means of observing aphid feeding behaviour patterns in the intact organism (Tjallingii 1978), and in the early 1980s, interest in the link between aphid behaviour and olfactory reception led to the development of two important methodologies: the electroantennogram technique, which measures the sum of receptor potentials from neurons close to the site of the recording electrode (van Emden and Harrington 2007; Wohlers and Tjallingii 1983; Park and Hardie 1998), and electrosensillograms, which measure olfactory receptor potentials directly from individual antennal sensilla (Nagai 1983; Wadhams 1982, 1984). Whilst these types of recordings have proved useful for understanding plant–host interactions and developing new means of aphid control, they are unsuitable for direct observation of the neurotoxic actions of insecticides with excitatory or channel-blocking actions.

Despite the devastating economic impact caused by aphids, no current electrophysiological method has been reported for the study of neurotoxicity in these insects. This is likely due to their relatively small size, which presents difficulties in studying their anatomy and neurophysiology. The giant willow aphid (*Tuberolachnus salignus*) is one of the world’s largest and longest-lived species of aphid reaching 4–5 mm when fully grown (Blackman and Eastop 1994). Its value for these studies is based on its larger size which facilitates dissection, although this species is itself an increasingly important economic pest (Collins et al. 2001a; Collins et al. 2001b). It also shows a susceptibility to aphicides that is very similar to more commercially significant pest species, thus providing a useful model for studying insecticide effects in aphids.

Imidacloprid (IMD) is a highly successful systemic insecticide which was the first of a class of chemicals called the neonicotinoids to be commercialised by Bayer in 1991 and as such is perhaps the most widely studied of its class. IMD has a very broad range of agricultural applications but is particularly effective against aphids, whitefly and hoppers hence its importance for control of sucking pests and the viral diseases for which these insects act as vectors. The neurotoxic action of neonicotinoids has been shown to be due to their ability to bind selectively and with nanomolar affinity to nicotinic acetylcholine receptors (Lind et al. 1998), a class of pentameric ligand-gated ion channels essential for fast synaptic transmission in the insect nervous system. For a comprehensive review of the neonicotinoids, their use and mode-of-action see Jeschke and Nauen (2010).

Here, we present a technique, using established extracellular recording methods enabling the neurotoxic effects of insecticides to be visualised directly in the exposed nervous system of an aphid.

## Methods

### Insects

The aphid colony was maintained on *Salix fragilis* (crack willow) saplings and originated from a stock kindly donated by Dr. C.M. Collins (Imperial College London, Silwood Park Campus). Willow twigs (25 cm lengths) with buds were placed in compost and allowed to grow for 3 weeks. Older plants were also sometimes trimmed to the correct size and used. The colony was maintained by laying pieces of infested willow from the eldest plants onto new plants. The insects were kept in a controlled environment cabinet (Sanyo MLR) set at 21 ± 2 °C, 60 ± 5 % humidity and 16/8 h light/dark cycles. The largest adults were chosen for experiments and allowed to feed right up to the point of experiment.

### Dissection

**Fig. 1:**
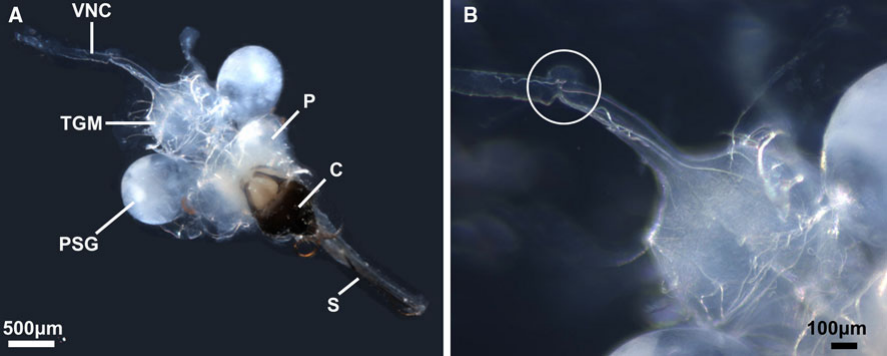
Dorsal view of the excised *T. salignus* CNS and associated salivary glands (**a**). Structures are labelled in accordance with previous anatomical descriptions (Hardie 1987b): ventral nerve cord (*VNC*), thoracic ganglionic mass (*TGM*), principal salivary gland (*PSG*), proctocerebrum (*P*), clypeus (*C*) and stylet (*S*). The *magnified portion* of the nerve cord **b** shows the typical position where the recording electrode is placed (*white circle*)

Apterous adult female *T. salignus* were gently pressed ventral side down onto double-sided sticky tape positioned centrally in a petri dish 1/3 filled with Sylgard^®^ resin (Dow Corning). Two dorsal incisions were made, one medial incision from the posterior to anterior of the abdomen and another mediolaterally, along the boundary between the thorax and abdomen. The resultant tergal flaps were carefully lifted back using fine forceps (Dumostar 5SF) and lightly pressed onto the surface of the tape to hold them in position. The ventral nerve cord (VNC) was exposed by removing the gut, ovaries and developing embryos. The VNC originating from beneath the principle salivary glands was identified as it emerges from the base of the thoracic ganglionic mass (TGM) (Fig. 1). The preparation was completely submerged under a physiological solution previously described by Caccia et al. (2005) containing (mM) MgSO_4_ 6, CaCl_2_ 2, KCl 13, MgCl_2_ 18, Na-citrate 2, KH_2_PO_4_ 12, sucrose 425, Hepes 10, Tris 5.9, and pH 6.4. The preparation was then transferred to the electrophysiology set-up and placed under gravity-fed continuous perfusion with a flow rate of approximately 1.0 ml min^−1^. The inflow was positioned directly above the TGM such that the emerging solution completely bathed the exposed internal structures of the preparation.

### Recording

Extracellular recordings of spontaneous activity were made using a suction electrode consisting of a borosilicate glass capillary (GC150F10, Harvard Apparatus) pulled to a sharp tip using a microelectrode puller (Sutter instruments company, model P-97). To ensure tight seals for each individual preparation, the tip was manually broken to a diameter of approximately 40 μm. The glass microelectrode was mounted over a silver wire (Teflon-coated 0.02 mm) that had been electroplated over approximately 2 mm at its tip to provide a Ag/AgCl exchange interface. This was fitted to a headstage (HS2A × 0.1L, Axon Instruments) connected to an amplifier (Axoclamp 2A, Axon Instruments). The reference electrode was placed in the bath containing the preparation. Using a small amount of back pressure applied from a syringe attached to the micropipette holder, a loop of the VNC at the position shown in Fig. 1 was drawn into the electrode. Once a tight seal was achieved, the electrode position was adjusted to provide some slack and avoid undue stress on the VNC.

The recording chamber was enclosed within a Faraday cage to reduce electrical noise interference. Amplified voltage signals were low-pass-filtered at a −3 dB cut-off frequency of 30 kHz and sent to a signal conditioner to provide an AC-coupled output signal. This was then filtered to remove power-line interference through a HumBug 50/60 Hz Noise Eliminator (Quest Scientific) and captured to computer via an Axon Digidata 1440 A interface (Molecular Devices Corp.) as a gap-free recording at a sample rate of 10 kHz.

### Chemicals

Imidacloprid (IMD) (synthesised by Syngenta) was dissolved in acetone to generate a stock solution (10 mM). Test solutions were prepared by diluting the stock solution into physiological saline to the final concentration. IMD was applied to the bath via the perfusion inflow. Acetone levels in the bath and test solutions were kept constant throughout the experiment at 0.1 %, a concentration which had no effect on spike frequency (data not shown).

### Analysis

Using Clampfit 10 software (Molecular Devices Corp.), the gap-free data file was loaded and filtered (1 kHz low pass) prior to analysis using one of the two alternative methods described below (threshold and template). The comparative advantages and disadvantages of these methods are described in the “Discussion”.

#### Threshold search

**Fig. 2:**
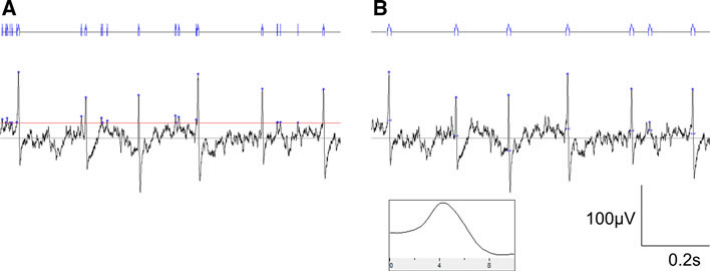
In a threshold search, all activity that crosses the 25-μV threshold line (*red*) is counted (**a**). Using a template search, spiking activity that matches the 10-ms template (*inset*) is counted (**b**)

The threshold search method detects events when the recorded signal crosses the trigger threshold value (Fig. 2a), which in our experiments was positioned 25 μV from the baseline. This value was established by analysing a 1-min region from 20 experiments where treatment with IMD had resulted in complete nerve block. Analysis of a 1-min region of each trace corresponding to complete block showed that the mean minimum distance from the baseline where no event crossing occurred was 25 μV.

The threshold value was set as a positive or negative value, depending on whether the majority of spikes showed greater peak amplitude in their positive-going or negative-going regions.

#### Template search

Templates were created by averaging 50 spikes from a 5-min sample region of the control region of each experiment. Electrical events that matched the template kinetics were then counted for analysis (Fig. 2b). These included spikes with varying amplitudes.

Following the spike counting procedure, the spike amplitude and the time of peak were used to construct scatter plots of amplitude and frequency histograms (1,000 ms bin), respectively, over experiment time.

Values for control spike frequency (events s^−1^) given in the results section and shown in Table 1 were calculated by averaging 5 min of activity. Statistics between template and threshold search analysis were calculated and compared using Student’s unpaired *t*-test. For experiments demonstrating the effect of IMD, spike frequency was calculated by averaging 30 s of activity before and during the application of the drug. The statistics were calculated using Student’s paired *t*-test. Results are presented as a mean ± SEM.

**Table 1:** Averaged frequency of spontaneous CNS spiking using template and threshold analysis for all experiments

	All experiments (*n* = 28 s^−1^)	Two populations (*n* = 22 s^−1^)	One population (*n* = 6 s^−1^)
Threshold search			
All amplitudes	43.3 ± 4.3*	42.6 ± 5.3*	46.0 ± 5.2*
Large amplitude	9.4 ± 0.7	9.6 ± 0.9	8.5 ± 1.1
Small amplitude	33.9 ± 4.2*	33.0 ± 5.1*	37.5 ± 5.3*
Template search			
All amplitudes	14.5 ± 1.4	16.3 ± 1.6	8.1 ± 1.1
Large amplitude	8.6 ± 0.6	8.8 ± 0.8	8.1 ± 1.1
Small amplitude	5.9 ± 1.2	7.5 ± 1.3	0

These were further divided into two categories: experiments where two distinct spike populations were present and those that showed one spike population using the template search

* *p* < 0.05 significance compared with template analysis

## Results

### Spontaneous CNS activity

**Fig. 3:**
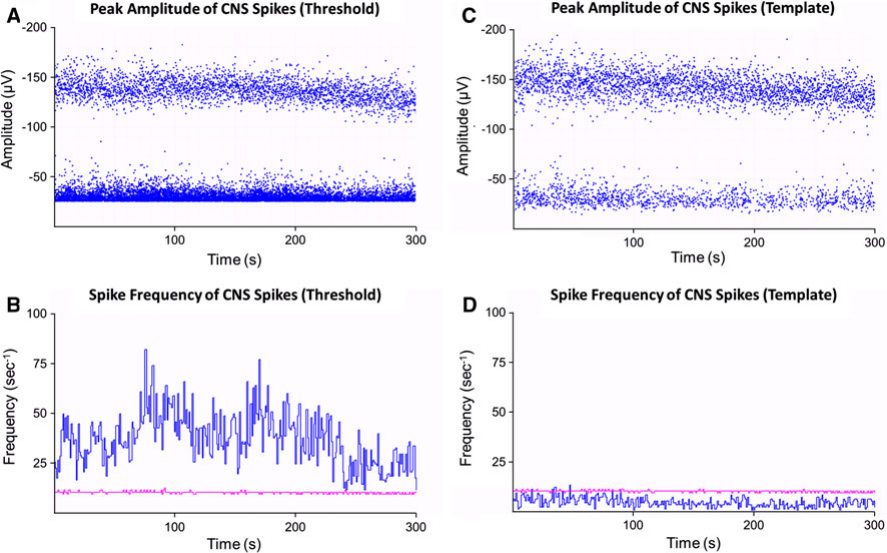
In most preparations, analysis using threshold (**a**, **b**) or template (**c**, **d**) methods revealed two distinct populations of peak spike amplitudes (**a**, **c**). Frequency histograms (**b**, **d**) revealed that large amplitude spikes (*pink*) were occurring at regular intervals, whilst smaller amplitude spikes (*blue*) had irregular frequencies

Spontaneous spiking activity was recorded in approximately 90 % of all preparations for the experimental duration of 1 h. In 28 experiments, CNS spiking occurred at a frequency of 43.3 ± 4.3 s^−1^ using the threshold search and 14.5 ± 1.4 s^−1^ using the template search (*p* < 0.05, *n* = 28, Table 1). The greatest difference between the two analytical methods is seen in the frequency of smaller amplitude spikes; 5.9 ± 1.2 s^−1^ (template) compared to 33.9 ± 4.2 s^−1^ (threshold) which impacts on the overall spike frequency. In more than 70 % of preparations, spikes were detected as two distinct amplitude populations (Fig. 3). Separate analysis of these two populations revealed that the larger amplitude spikes were occurring at a more steady frequency of 8.8 ± 0.8 s^−1^ (template search, Table 1) and 9.6 ± 0.9 s^−1^ (threshold search, *p* > 0.05, *n* = 22, Table 1). The smaller amplitude spikes had varying frequencies, occurring at 7.5 ± 1.3 s^−1^ using the template search analysis and 33.0 ± 5.1 s^−1^ with threshold analysis (*p* < 0.05, *n* = 22, Fig. 3). In six experiments, the spikes recorded with the template search could not be separated into varying amplitude populations and hence were regarded as one population. However, in these experiments, two populations could still be resolved using the threshold search method of analysis (Table 1).

### Effects of imidacloprid

Analysis using threshold search showed that the neonicotinoid, IMD (1 μM) initially increased spiking frequency by approximately 50 %, from 36.3 ± 6.4 s^−1^ to 54.5 ± 7.6 s^−1^ (*p* < 0.05, *n* = 4, Table 2). This change occurred in the smaller amplitude spike population, as the frequency of the larger spikes during this period remained unchanged from 8.5 ± 1.5 s^−1^ to 8.7 ± 2.2 s^−1^ (*p* > 0.05, *n* = 4, Table 2). Two minutes after the initial application of IMD, the frequency of the smaller amplitude spikes was reduced to 4.4 ± 3.3 s^−1^ and larger spikes were abolished (*p* < 0.05, *n* = 4). These effects were irreversible after 20 min of washout.

**Table 2:** Effects of imidacloprid (1 μM) on the frequency of spontaneous CNS spiking using template and threshold analysis

	Control (s^−1^)	Imidacloprid (1 μM) initial (s^−1^)	Imidacloprid (1 μM) after 2 min (s^−1^)
Threshold search			
All amplitudes	36.3 ± 6.4*	54.5 ± 7.6*^,^^	4.4 ± 3.3^^^
Large amplitude	8.5 ± 1.5	8.7 ± 2.2	0^
Small amplitude	27.8 ± 7.3*	45.8 ± 7.6*^,^^	4.4 ± 3.3^^^
Template search			
All amplitudes	12.6 ± 2.3	15.0 ± 2.7^^^	2.1 ± 1.3^^^
Large amplitude	6.5 ± 1.5	5.6 ± 1.8	0^
Small amplitude	6.1 ± 1.8	9.4 ± 1.3^^^	2.1 ± 1.3^^^

* *p* < 0.05 significance compared with template analysis; ^ *p* < 0.05 significance compared with control (*n* = 4)

**Fig. 4:**
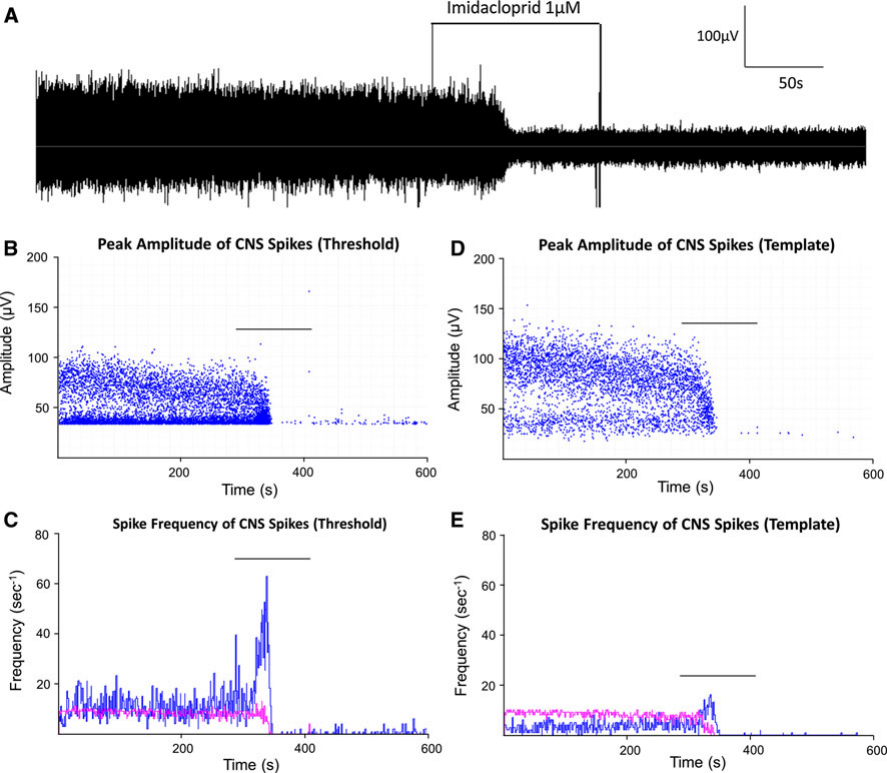
The raw data trace demonstrated that IMD (1 μM) rapidly abolished spiking activity in the aphid CNS (**a**). Threshold and template analysis was used to create amplitude *scatter plots* (**b**, **d**) and frequency histograms. The histograms show the frequency of small (*blue*) and large (*pink*) amplitude spikes over the course of the experiment (**c**, **e**). *Bars* in graphs represent the application of IMD

The effects of IMD on CNS spiking activity appear similar whether template or threshold search analyses are used (*n* = 4, Fig. 4, Table 2). Template analysis showed that within 30 s, IMD caused a slight increase in spike frequency from 12.6 ± 2.3 s^−1^ to 15.0 ± 2.7 s^−1^ (*p* < 0.05, *n* = 4, Table 2), although the frequency of larger spikes remained unchanged from 6.5 ± 1.5 s^−1^ to 5.6 ± 1.8 s^−1^ (*p* > 0.05, *n* = 4). After 2 min, these larger spikes were abolished, whilst the smaller spikes were still occurring at 2.1 ± 1.3 s^−1^ (*p* < 0.05, *n* = 4, Table 2). The results were similar with the threshold method except that the initial excitation response in the small spike population was more obvious: 27.8 ± 7.3 s^−1^ to 45.8 ± 7.6 s^−1^ (threshold) compared with 6.1 ± 1.8 s^−1^ to 9.4 ± 1.3 s^−1^ (template, Fig. 4).

## Discussion

To our knowledge, this is the first study to report spontaneous spiking activity from the VNC of an aphid species. The extracellularly recorded activity in the VNC reveals at least two populations of spikes, distinguishable by their amplitude. The large amplitude population exhibits a regular ‘pacemaker’ type frequency, whilst the small amplitude spikes are more irregular in nature. Steady rhythmical elements have been noted in extracellular CNS recordings of other invertebrates such as *Drosophila* (Fox et al. 2006) and locust (Ayali et al. 2002), but rather than simple tonic firing, these have tended to consist of more complex burst patterns characteristic of central pattern generators (for review see Selverston 2010).

The neonicotinoid, IMD (1 μM) caused a blockade of spontaneous spiking activity in this aphid preparation that was commonly preceded by an elevation in spike frequency. A convenient explanation for this effect is that IMD acts initially as an agonist to produce a depolarisation at the postsynaptic membranes of cholinergic synapses resulting in a transient excitation. This later gives way to blockade as the compound accumulates in the nervous tissue, reaching concentrations at which receptor desensitisation becomes the dominant effect. Similar patterns of excitation and blockade in response to IMD have previously been reported for cockroach CNS preparations (Buckingham et al. 1997). However, the recorded response to IMD in this aphid preparation is likely to be the net product of numerous interactions with nicotinic receptors of differing receptor subunit identity and stoichiometry. IMD is known to have diverse interactions across a range of nerve preparations that range from partial agonist to antagonist behaviour depending on concentration and cell (receptor)-type (Matsuda et al. 2009). It is therefore entirely possible that a combination of agonist, agonist-induced desensitisation and antagonist actions are contributing to the overall pattern of activity observed.

Homopterans have been shown to possess two high affinity binding sites for IMD, the aphid *Myzus persicae* having dissociation constants of 0.14 and 12.6 nM, respectively (Lind et al. 1998). Concentrations at which activity is seen in neurophysiology preparations are typically two to four orders of magnitude higher than corresponding binding affinities might suggest (Nauen et al. 2001; Jeschke and Nauen 2010; Buckingham et al. 1997; Lind et al. 1998; Salgado and Saar 2004). This could be due to a number of factors. Nicotinic receptors in membrane preparations exist in a depolarised, desensitised state in which they typically exhibit higher agonist binding affinity than when in the resting state (Léna and Changeux 1993). Furthermore, in this particular preparation, the neurones are not directly exposed to the applied concentration. The compound must still circumvent the perineurial sheath layer and diffuse to its binding site, so application of the compound at higher than threshold concentration is necessary to see manifestation of a response in a time frame that is suited to the experiment.

Whilst both the threshold and template analysis methods are clearly capable of detecting the actions of IMD (Fig. 4), the errors and anomalies within the data differ between the two. The threshold method will cleanly count fast signals of amplitude higher than the threshold, but events which peak close to the threshold level may be detected as several crossings if the background noise becomes superimposed on the level of the event discriminator. As a consequence, a greater number of small amplitude events are counted, affecting the overall spike frequency (Figs. 3, 4). The template method overcomes this problem as spike recognition is based on fit to a kinetic profile. However, more of the smaller amplitude spikes are missed because the reduction in their size relative to the background noise brings with it an associated increase in probability that the noise will sufficiently perturb their kinetic profile to prevent their recognition by fit to the template. We have included both methods in this article because either might yet prove more suitable than the other for studying the subtle effects of particular drugs or changes in the environmental conditions of the preparation, which are the focus of ongoing work.

We have considered whether the large amplitude activity of regular frequency could be contributed by a single axon in this aphid ventral nerve cord. Single axons can dominate an extracellular nerve recording where they account for a significant proportion of the overall diameter of the nerve because the amplitude of the extracellular action potential varies directly with cell diameter. In the case of the cockroach giant axon (Yamasaki and Narahashi 1959) or cercal giant interneuron (Daley et al. 1981), the larger axons have been evident in micrographs of transverse sections of the nerve (Meiri et al. 1983; Blagburn and Beadle 1982). However, no such ‘giant’ axons were revealed by electron microscopy studies of transverse sections of nerve cord posterior to the VNC in the aphid *Megoura viciae* (Jim Hardie, unpublished communication). The observation that the larger amplitude spike population did not increase initially in response to IMD may suggest a difference in the way these spikes are propagated or regulated relative to the smaller spikes. However, the physiological origin and significance of these different populations are currently unknown, hence further studies to examine their pharmacological properties is still required.

It is reasonable to assume that the perineurial layer that surrounds the nervous system remains intact and functional in this CNS preparation. Aphid haemolymph is unusual amongst insects in that the concentration of sodium ions it contains is the lowest recorded for any animal (0.2–2 mM) (Downing 1980). Whilst the actual inorganic ion concentrations of the haemolymph of *T. salignus* have not been reported, we found in our experiments that the greatest success in recording action potentials was achieved in the saline of (Caccia et al. 2005) in which values were in turn based on published values for hemolymph of *Aphis fabae* (Hardie 1987a), *Myzus persicae* (Downing 1980) and *Macrosiphum* *albifrons* (Pelletier and Clark 1995). The low concentration of sodium (2 mM) and calcium (2 mM) is unlikely to support conventional action potentials unless highly effective ion-exchange barriers are in place to concentrate these ions in the neuronal microenvironment. Very little is currently known about how this is achieved. Anatomical studies of *Megoura viciae* identified neural lamellae surrounding a tracheolated perineurial layer in the VNC close to the base of TGM, and a perineurial sheath was also evident in minor nerves emanating from the dorsal TGM, but interestingly, axons in abdominal visceral nerves appeared to be separated from the hemolymph only by the neural lamella (Hardie 1987b). Ultrastructural studies of intrinsic neurosecretory cells of the corpora cardiaca of *Myzus persicae* showed a similar absence of glial protection (Bowers and Johnson 1966). Clearly, much further study is required to understand the structures and physiological processes responsible for regulation of excitability in aphid axons and neurosecretory cells.

The described method provides a technique for assessing the neurotoxicity of chemicals directly in the central nervous system of a previously intractable pest family. This technique should prove to be of great benefit in the development of new insecticidal compounds targeted to aphids and provides a tool for research into the mechanisms by which central nervous function is maintained in a low solute environment.
